# C_4_ Phosphoenolpyruvate Carboxylase: Evolution and transcriptional regulation

**DOI:** 10.1590/1678-4685-GMB-2023-0190

**Published:** 2024-03-22

**Authors:** Pedro Carvalho, Célia Gomes, Nelson J.M. Saibo

**Affiliations:** 1 Universidade Nova de Lisboa, Instituto de Tecnologia Química e Biológica António Xavier, Oeiras, Portugal.

**Keywords:** C_4_ photosynthesis, transcriptional regulation, PEPC, C_3_ to C_4_ evolution

## Abstract

Photosynthetic phosphoenolpyruvate carboxylase (PEPC) catalyses the irreversible carboxylation of phosphoenolpyruvate (PEP), producing oxaloacetate (OAA). This enzyme catalyses the first step of carbon fixation in C_4_ photosynthesis, contributing to the high photosynthetic efficiency of C_4_ plants. PEPC is also involved in replenishing tricarboxylic acid cycle intermediates, such as OAA, being involved in the C/N balance. In plants, PEPCs are classified in two types: bacterial type (BTPC) and plant-type (PTPC), which includes photosynthetic and non-photosynthetic PEPCs. During C_4_ evolution, photosynthetic PEPCs evolved independently. C_4_ PEPCs evolved to be highly expressed and active in a spatial-specific manner. Their gene expression pattern is also regulated by developmental cues, light, circadian clock as well as adverse environmental conditions. However, the gene regulatory networks controlling C_4_ *PEPC* gene expression, namely its cell-specificity, are largely unknown. Therefore, after an introduction to the evolution of PEPCs, this review aims to discuss the current knowledge regarding the transcriptional regulation of C_4_ PEPCs, focusing on cell-specific and developmental expression dynamics, light and circadian regulation, as well as response to abiotic stress. In conclusion, this review aims to highlight the evolution, transcriptional regulation by different signals and importance of PEPC in C_4_ photosynthesis and its potential as tool for crop improvement.

## Phosphoenolpyruvate carboxylase in plants and its rise to power

Phosphoenolpyruvate carboxylase (PEPC; EC 4.1.1.31) is a ubiquitous and cytosolic enzyme, responsible for the irreversible β-carboxylation of phosphoenolpyruvate (PEP), in the presence of HCO_3_^-^, producing oxaloacetate and inorganic phosphate (Pi) (O’Leary, 1982; Chollet et al., 1996; O’Leary *et al.*, 2011). It can be found in non-photosynthetic bacteria, cyanobacteria, green algae, and in all land plants (O’Leary *et al.*, 2011).

**Figure 1 -:**
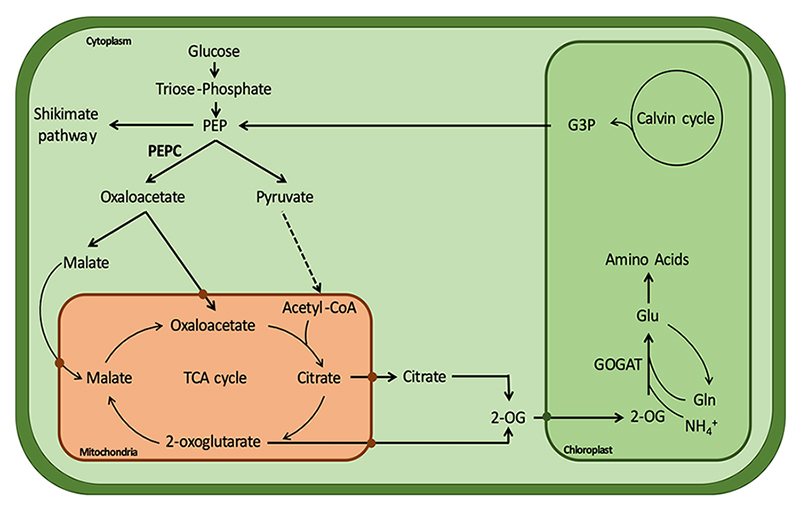
Simplified schematic representation of the role played by non-photosynthetic PEPC in the carbon-nitrogen balance. The carboxylation of phosphoenolpyruvate (PEP) is an important step to replenish carbon skeletons to the TCA cycle, re-routing carbon (glycolysis products) into the TCA cycle. The link between the TCA and GOGAT/GS cycles is important for the carbon-nitrogen balance, making PEPC an important regulator of carbon partitioning.

In most organisms, PEPC plays an anaplerotic role being important to replenish intermediates, namely oxaloacetate, in the tricarboxylic acid cycle, by re-fixing the CO_2_ released by respiration, thus allowing an increased flux throughout this cycle (Sánchez and Cejudo, 2003). In plants, it occupies a central place in the primary carbon metabolism, linking the carbon and nitrogen metabolism (Figure 1) (O’Leary et al., 2011). In *Arabidopsis thaliana*, plants lacking PEPC1 and PEPC2 show growth arrest in control conditions, which is linked to a disrupted carbon-nitrogen balance. Double mutants not only show reduction of NH_4_^+^ fixation, by repression of the GOGAT/GS cycles, but also an accumulation of sucrose and starch granules in the chloroplasts, having impaired starch degradation (Shi et al., 2015).

In C_4_ and CAM plants, one of the PEPCs playing an anaplerotic role evolved to have a role in photosynthesis. For these plants, the irreversible carboxylation performed by PEPC is the first step of carbon assimilation, being therefore a key enzyme for the proper operation of C_4_ and CAM photosynthesis. Since it is possible to distinguish between their anaplerotic and photosynthetic roles, plant PEPC isoforms are divided into photosynthetic and non-photosynthetic (O’Leary et al., 2011).

### PEPCs in plant genomes

**Figure 2 -:**
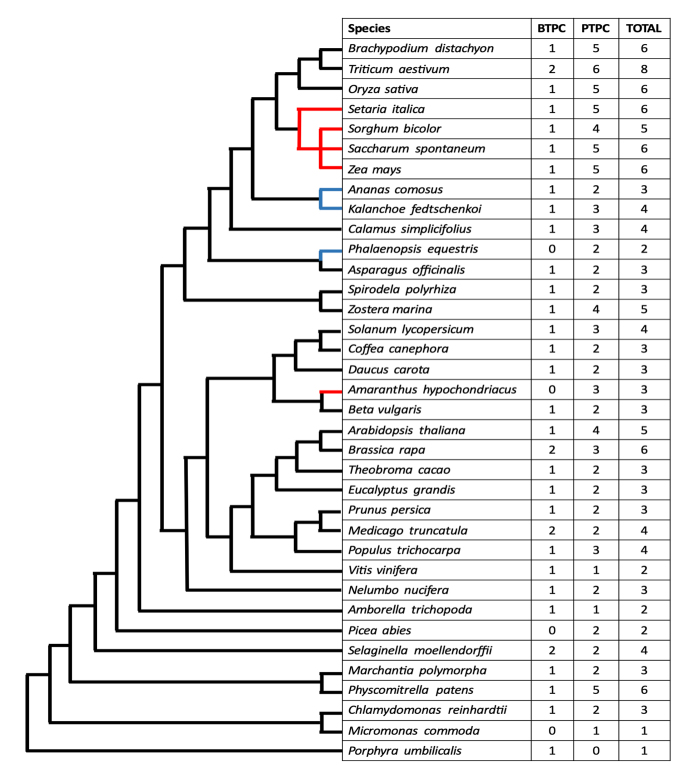
Cladogram representing the amount of PEPC isoform present in plant genomes. Species are organised considering their phylogenetic relationships, with representatives of important evolutionary groups. Sequences were obtained from PLAZA and NCBI databases, using different PEPC protein sequences for BLASTp. Incomplete or unrelated sequences were removed by protein alignment and phylogenetic analysis. Red lines represent C_4_ species, blue lines represent CAM species, and black lines represent C_3_ species.

In plants, the different PEPC enzyme isoforms are encoded by a small multigene family. Within this family, two major lineages can be distinguished: bacterial-type (BTPC) and plant-type (PTPC) PEPCs (O’Leary et al., 2011). At least one copy of the *BTPC* gene can be found in most plant species sequenced to date (Figure 2). BTPCs found in both dicots and monocots are phylogenetically closer to PEPCs from bacteria than to PTPCs (O’Leary *et al.*, 2011). In addition to its different gene structure, BTPCs and bacterial PEPCs lack a N-terminal Serine residue, which can be phosphorylated, an important feature that distinguishes them from PTPCs (Sánchez and Cejudo, 2003). It has been proposed that when Viridiplantae (green plants) arose, PTPC originated from BTPC through gene duplication (Chang et al., 2013). 

Plant-type PEPCs typically can be found as homotetramers and traditionally they are divided as photosynthetic, for those involved in C_4_ or CAM photosynthesis, or non-photosynthetic, PTPCs not involved in photosynthesis in either C_3_ or C_4_ species. Although diverse, all plant PEPCs are thought to have appeared from a single ancestral form (Svensson et al., 2003). 

### The path to C_4_ photosynthesis

To overcome the energy loss due to photorespiration, a process that metabolises a toxic compound generated when Rubisco acts as oxygenase, some plants have evolved a carbon concentration mechanism called C_4_ photosynthesis. In most C_4_ plants, CO_2_ is first fixed in the mesophyll cells by PEPC, into a four-acid compound that is shuttled to the bundle sheath cells where it is decarboxylated, thus increasing the CO_2_ concentration around Rubisco. In addition to the two-cell type C_4_ photosynthesis, a few plants have developed C_4_ photosynthesis in a single-cell, where the spatial separation of the carbon fixation reactions occurs inside one cell. For instance, in the single-cell C_4_ species *Bienertia sinuspersici*, C_4_ photosynthesis is based on an intracellular compartmentation including two physiologically and biochemically different chloroplast types (Caburatan et al., 2019). Evolution of C_4_ photosynthesis has occurred over 60 independent times, in both dicotyledons and monocotyledons, in one of the most amazing examples of convergent evolution known in nature (Sage *et al.*, 2011). Despite the broad evolutionary trajectories of C_4_ photosynthesis, all C_4_ species rely on PEPC for the first carboxylation step (Sage *et al.*, 2011). Many authors have tried to resolve the evolutionary origin of PEPCs and they have clearly shown that photosynthetic C_4_ PEPCs from dicots and monocots evolved from different C_3_ origins (Westhoff and Gowik, 2004; Christin et al., 2007; Besnard et al., 2009; Christin and Besnard, 2009).

In the dicot *Flaveria* genus, which contains C_3_, C_4_ and C_3_-C_4_ intermediate species, it is possible to distinguish 3 classes of PEPC genes (A, B, and C) (Westhoff and Gowik, 2004). PEPCs from class A are present in both C_3_ and C_4_ species and class A C_4_ PEPCs originated from a duplication of class B PEPCs. The photosynthetic PEPCs belong to class A and originated from a duplication of class B PEPCs. Class A C_4_ PEPCs (ppcA) are present in both C_3_ and C_4_ species, however, although these genes show variable transcript levels among species, in C_4_-like intermediate species, ppcA transcript levels are higher and similar to C_4_ plants (Engelmann et al., 2003). Therefore, C_4_ PEPC isoforms seem to have evolved in a stepwise fashion, with the increase of gene expression preceding amino acid changes (Westhoff and Gowik, 2004; Engelmann *et al.*, 2003).

In the clade PACMAD (named based on its subfamilies Panicoideae, Aristidoideae, Chloridoideae, Micrairoideae, Arundinoideae, Danthonioideae), which comprises all the grass C_4_ species, PEPCs have evolved over eight independent times, recruiting different C_3_ PEPC isoforms to acquire the C_4_ function (Christin et al., 2007; Christin and Besnard, 2009). In most grass species, the recruited isoform was *ppc-B2*, while in the case of *Stipagrostis* genus, it was *ppc-A1b* isoform (Christin and Besnard, 2009). In the case of sedges (Cyperaceae), the PEPC isoform recruited for C_4_ photosynthesis is sister of the *ppc-A1a* and *ppc-A1b* isoforms from grasses, evolving five independent times (Besnard *et al.*, 2009; Christin and Besnard, 2009).

It is yet to be defined which amino acid changes are responsible for the evolution from a C_3_ to a C_4_ isoform. Despite some amino acid positions having been proposed as being under positive selection for C_4_ function (Christin et al., 2007), only one amino acid substitution has been conclusively linked to the C_4_ isoform of PEPC (Bläsing et al., 2000). The substitution of an Alanine to a Serine can be found in C_4_ PEPCs of several dicots and monocots, making it a key criterion for C_4_ isoform definition. It occurs in position 780 in maize (Christin *et al.*, 2007), and 774 in *Flaveria* species and significantly influences PEPC kinetic properties (Bläsing *et al.*, 2000). Besides the specific protein features, PEPC transcriptional regulation in C_4_ plants is tightly controlled and its essential for the proper functioning of C_4_ metabolism.

## Transcriptional regulation of C_4_ PEPC

### Developmental regulation

In monocots and dicots, leaves differentiate following a gradient, in which younger cells are present at the leaf base, while older and more mature cells are present at the leaf tip (Nelson and Langdale, 1989; Stockhaus et al., 1997; Aubry et al., 2014). During leaf development, C_4_ *PEPC* gene expression is regulated by developmental cues, increasing gradually from leaf base to leaf tip (Martineau and Taylor, 1985; Stockhaus *et al.*, 1997; Pick et al., 2011; Aubry *et al.*, 2014; Tao et al., 2022). In maize and *Cleome gynandra*, C_4_ *PEPC* transcript level is higher in mature than in younger leaves (Kausch et al., 2001; Aubry *et al.*, 2014). Since mature leaves have more differentiated M cells than younger leaves, it seems that C_4_ *PEPC* expression level follows M cells differentiation. In fact, maize *PEPC* was recently identified as a target of COL8, a transcription factor (TF) co-regulated with *PEPC* during M cell development (Tao *et al.*, 2022). This suggests that COL8 might regulate *PEPC* expression during leaf development, however further investigation is required to validate this TF as a *PEPC* gene regulator. A developmental regulation of C_4_ *PEPC* gene expression was also observed in the single-cell type C_4_ species *Bienertia sinuspersici.* In this species, gene expression analysis of PEPC isoforms showed that C_3_ *PEPC* is more expressed in the younger leaves or early stages of development, while C_4_ *PEPC* is upregulated in the mature stages of leaf development (Caburatan et al., 2019). However, C_4_ *PEPC* gene expression does not follow a developmental pattern in all species. In the particular case of Amaranth, C_4_ *PEPC* is highly expressed since the beginning of leaf development, namely in leaf primordia and in the apical meristem and surrounding regions (Ramsperger et al., 1996).

C_4_ PEPC protein accumulates at different leaf development stages in a species-dependent manner (Mayfield and Taylor, 1984; Martineau and Taylor, 1985; Dengler et al., 1995; Soros and Dengler, 2001; Voznesenskaya et al., 2003; Wakayama et al., 2003; Majeran et al., 2010; Koteyeva et al., 2014) and, in general, C_4_ PEPC accumulation goes along with M cells differentiation (Voznesenskaya *et al.*, 2003; Wakayama *et al.*, 2003; Majeran *et al.*, 2010; Koteyeva *et al.*, 2014). Nevertheless, the mechanisms coordinating C_4_ *PEPC* gene expression and protein accumulation during leaf development differ among species (Langdale et al., 1988; Wang et al., 1992; Wang *et al.*, 1993; Dengler *et al.*, 1995; Ramsperger et al., 1996; Soros and Dengler, 2001; Voznesenskaya *et al.*, 2003; Wakayama *et al.*, 2003; Koteyeva *et al.*, 2014). In the case of amaranth, in early developmental stages, C_4_ *PEPC* gene expression does not occur in a cell-specific way, however, the expressed protein is only present in the M cell precursors (Ramsperger *et al.*, 1996). This pattern is also observed in cotyledons and maintained in later stages of leaf development, namely during leaf unfolding (Wang *et al.*, 1992; Wang *et al.*, 1993). Although no information is available regarding the regulatory mechanisms underlying C_4_ *PEPC* gene expression in amaranth during leaf development, post-transcriptional or translational regulation mechanisms seem to play the main role in regulating cell-specific C_4_ PEPC protein accumulation (Wang *et al.*, 1992; Wang *et al.*, 1993; Ramsperger *et al.*, 1996). In contrast, maize C_4_ *PEPC* is expressed in a cell-specific way throughout leaf development (Langdale *et al.*, 1988; Majeran *et al.*, 2010). Hence, transcriptional regulatory mechanisms seem to be the most important to establish a C_4_ *PEPC* cell-specific expression pattern in maize. Other species known to accumulate C_4_ PEPC only in M cells, regardless of developmental stage, are *Atriplex rosea*, *Arundinella hirta* and two *Cleome* species (Dengler *et al.*, 1995; Wakayama *et al.*, 2003; Koteyeva *et al.*, 2014), however, the regulatory mechanisms underlying this feature are not known. A different example is *Salsola richteri*, in which C_4_ PEPC protein starts to accumulate in a non-cell specific way at early stages, being present in BS and M cells, and other leaf cells albeit at lower levels, but, in later stages of leaf development, C_4_ PEPC is detected exclusively in M cells (Voznesenskaya *et al.*, 2003). The mechanisms regulating *S. richteri* C_4_ PEPC cell-specific accumulation are also unknown. Similarly to *Salsola richteri*, in two Cyperaceae species, *Pycreus polystachyos* and *Eleocharis retrofiexa*, C_4_ PEPC accumulation only becomes cell-specific later in leaf development (Soros and Dengler, 2001). In *Eleocharis retrofiexa* C_4_ PEPC accumulation is also present in the parenchymatous BS (PBS), suggesting that PBS and M cells have similar functions (Soros and Dengler, 2001). In the particular case of *Rhynchospora rubra*, another Cyperaceae species, C_4_ PEPC never accumulates in a cell-specific way throughout leaf development, suggesting that *Rhynchospora rubra* may have a different version of C_4_ photosynthesis (Soros and Dengler, 2001). Although these three species belong to the same family, the differences regarding C_4_ PEPC accumulation may be related to the different C_4_ origins they represent and to the differences in the anatomical features between species (Soros and Dengler, 2001).

The fact that C_4_ *PEPC* gene expression and protein accumulation patterns during leaf development differ among species shows that different species acquired different developmental regulatory mechanisms during C_4_ evolution, which is not surprising given the evolutionary convergence of C_4_ photosynthesis. To better understand these regulatory mechanisms, more information regarding C_4_ *PEPC* transcriptional regulation in different species during their leaf development is needed. 

### Spatial regulation

In most C_4_ plants, photosynthetic reactions are divided into two different cell types, M and BS cells. As stated in section 1b, C_4_ PEPC first fixes CO_2_ in M cells, where it is highly and specifically expressed (Sage, 2004). This expression pattern required the development of a complex regulatory network during C_4_ evolution. It has been suggested that the transcriptional mechanisms regulating non-photosynthetic *PEPC* gene expression were modified to reach a high and cell-specific transcript level (Williams et al., 2012). The recruitment of *cis*-elements and TFs regulating C_3_ genes was essential to achieve this purpose (Williams *et al.*, 2012).

**Figure 3 -:**
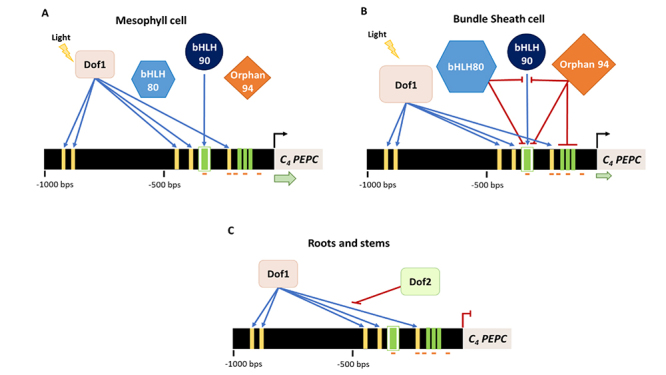
Schematic representation of the different mechanisms proposed to regulate the transcription of C_4_ *ZmPEPC* in an organ- and cell-specific way. (A) Regulation of C_4_ *ZmPEPC* gene expression in M cells. The repressors ZmbHLH80 and ZmOrphan94 are less expressed than in BS cells, therefore there is a high gene expression activation by ZmbHLH90. (B) Regulation of C_4_ *ZmPEPC* gene expression in BS cells. ZmbHLH80 and ZmOrphan94 are preferentially expressed in BS cells, working as repressors of ZmbHLH90, leading to a down-regulation of C_4_ ZmPEPC expression. ZmbHLH80 and ZmOrphan94 can impair ZmbHLH90 function through heterodimerization or competitive binding for the same binding site. In addition, ZmOrphan94 may also impair ZmbHLH90 through its binding to CACA motifs, close to ZmbHLH90 binding site. In leaves, Dof1 is activated by light, allowing its binding and consequent activation of C_4_ *ZmPEPC* gene expression (A and B). (C) Regulation of C_4_ *ZmPEPC* in stems and roots by Dof1 and Dof2. These TFs are both expressed in these tissues, however, while Dof1 bind to the respective cis-elements in the C_4_ *ZmPEPC* promoter to activate gene expression, Dof2 binds them to block Dof1 DNA-interaction, thus impairing C_4_ *ZmPEPC* expression. The black arrows and the red lines represent activation and repression of gene expression, respectively. The thickness of the green arrow represents the expression levels of C_4_ *PEPC* in each cell type. Activation and repression by the different TFs are represented as blue arrows and red lines, respectively. The different sizes of Dof1, ZmbHLH80 and ZmOrphan94, between A and B denote their gene expression levels in each cell type. The yellow rectangles represent the binding sites of Dof1 and Dof2 (Yanagisawa and Sheen, 1998) and the green rectangles represent the ZmOrphan94 binding sites. The binding site of ZmbHLH80 and ZmbHLH90 (E-box) is represented by a white rectangle. Within this E-box, there is a CACA motif, which is represented by a green rectangle, similar to the other binding sites of ZmOrphan94. The orange lines underneath the promoter represent the CNSs identified by Gupta et al. (2020).

In maize, the C_4_ *PEPC* promoter (C_4_ *ZmPEPC* promoter) drives a leaf-specific expression. Despite some gene expression in some leaf-like organs, the C_4_ *ZmPEPC* promoter shows a very high activity in leaves as compared with other mature tissues, such as roots and stems, in which no activity is detected (Kausch et al., 2001). Dof1 and Dof2 are two TFs identified as putative regulators of C_4_ *PEPC* organ-specific gene expression in maize (Yanagisawa and Sheen, 1998) (Figure 3). Dof1 is a ubiquitously expressed TF, working as a light-dependent activator, while Dof2 is only expressed in roots and stems and acts as a repressor (Yanagisawa and Sheen, 1998). *In vivo* experiments demonstrated that when Dof2 is expressed, it binds to the C_4_ *PEPC* promoter, impairing Dof1 binding and consequently promoter activation (Yanagisawa and Sheen, 1998). Therefore, it was hypothesised that, in stems and roots, Dof2 binds to the C_4_ *PEPC* promoter, blocking Dof1 DNA interaction and, consequently, down-regulating C_4_ *PEPC* transcript levels in these tissues (Figure 3A). In leaves, Dof1 is free to bind to the C_4_ *PEPC* promoter, thus activating it (Figures 3B and 3C) (Yanagisawa and Sheen, 1998). However, contrasting with this hypothesis, the knockout of *Dof1* does not affect C_4_ *PEPC* expression levels, implying that this TF does not have a prominent role in C_4_ *PEPC* transcriptional regulation (Cavalar et al., 2007). Another possibility is the existence of transcriptional redundancy by other Dof TFs or even TFs from other families. If this is true, the knockout of *Dof1* may not be sufficient to affect C_4_ *PEPC* expression levels. Hence, the identification of other TFs regulating C_4_ *PEPC* gene expression will be useful to understand how TFs regulate C_4_ *PEPC* expression in a tissue-specific way.

Recently, three additional maize TFs, ZmbHLH80, ZmbHLH90, and ZmOrphan94 have been identified as putative regulators of C_4_ *PEPC* cell-specific gene expression, having binding sites in the promoter regions known to be crucial to establish this expression pattern (Górska et al., 2019; Gupta et al., 2020; Górska *et al.*, 2021) (Figures 3A and 3B). ZmbHLH90 was shown to act as an activator of C_4_ *ZmPEPC,* while ZmbHLH80 and ZmOrphan94 act as repressors (Górska *et al.*, 2019; Górska *et al.*, 2021). It was proposed that both repressors, ZmbHLH80 and ZmOrphan94, play an important role in C_4_ *PEPC* cell-specific gene expression keeping its expression low in the BS cells, where they are preferentially expressed. The high *ZmbHLH80* and *ZmOrphan94* gene expression in the BS cells may lead to the formation of heterodimers with the activator ZmbHLH90, thus impairing its function (Górska *et al.*, 2019; Górska *et al.*, 2021) (Figure 3B). In M cells, ZmbHLH80 and ZmOrphan94 are less expressed and, therefore, ZmbHLH90 is free to form homodimers and thus activate C_4_ *ZmPEPC* expression (Górska *et al.*, 2019; Górska *et al.*, 2021). We must however emphasise that, though it was clearly shown that ZmbHLH80 and ZmOrphan94 transcript levels are higher in BS as compared with M cells, nothing is known about their protein abundance. In addition to the negative regulation by heterodimerization, we may have other regulation mechanisms between activators and repressors, such as competition for the same binding site, interaction after DNA binding or a stronger regulatory effect of repressors over activators (Górska *et al.*, 2021) (Figure 3). It would be interesting to investigate whether these new identified TFs interact with the TFs previously identified and, if they interact, how they function to regulate C_4_ *PEPC* gene expression. One could also hypothesise that a double mutant Dof1/ZmbHLH90 might be needed to affect C_4_ *ZmPEPC* gene expression.

In addition to TFs, *cis*-elements in the C_4_ *PEPC* promoter have also been associated with the mesophyll cell-specific gene expression (Gowik et al., 2004; Akyildiz et al., 2007; Gupta et al., 2020). Interestingly, it has been reported that C_4_ *PEPC* promoter regions underpinning cell-specific expression are different between dicots and monocots (Gowik *et al.*, 2004; Akyildiz *et al.*, 2007; Engelmann et al., 2008; Gupta *et al.*, 2020). In dicots, such as *Flaveria* species, a region of the distal promoter (2141 to 1566 bps before ATG) of C_4_ *PEPC* is responsible to establish the spatial expression pattern, while the proximal promoter region (570 bps before ATG) works as an enhancer of C_4_ *PEPC* expression, being both necessary for high and cell-specific expression levels (Gowik *et al.*, 2004; Akyildiz *et al.*, 2007; Engelmann et al., 2008). When the C_4_ *PEPC* proximal promoter region was isolated, no cell-specificity was observed. On the other hand, when the proximal promoter region was replaced by its C_3_ counterpart, although cell-specificity was maintained a decrease in promoter strength was observed (Gowik *et al.*, 2004; Akyildiz *et al.*, 2007; Engelmann et al., 2008). Although some *cis*-elements have been identified as putative enhancers within the proximal promoter, their role in C_4_ *PEPC* expression was never proven (Engelmann *et al.*, 2008). Deletions in the distal promoter, however, showed that a *cis*-element designated mesophyll expression module 1 (MEM1) is essential for a cell-specific expression. Without this element, or when it is replaced by its C_3_ counterpart, the M cell specificity is lost (Gowik *et al.*, 2004; Akyildiz *et al.*, 2007). In contrast to *Flaveria* species, the C_4_ *PEPC* proximal promoter (~500 bps) from grasses (monocots) is sufficient to drive a high M cell-specific expression, thus having all the necessary *cis*-elements to achieve cell-specificity (Schaffner and Sheen, 1992; Taniguchi et al., 2000; Gupta *et al.*, 2020). Within this region, four conserved nucleotide sequences (CNSs) were identified as essential *cis*-elements for an M cell-specific expression (Gupta *et al.*, 2020). When the CNSs were eliminated from the C_4_ *PEPC* promoter, the promoter activity was almost eliminated, being rescued when the original CNSs were replaced by equivalent sequences from a different C_4_ grass species (Gupta *et al.*, 2020).

In addition to the *cis* and *trans* factors, some epigenetic modifications might be involved in C_4_ *PEPC* gene expression regulation. Tri-methylation (H3K4me3) and di-methylation (H3K4me2) states, found in C_4_ *PEPC* proximal promoter and transcribed regions, seem to be associated with the establishment of C_4_ *PEPC* cell-specific expression (Danker et al., 2008; Heimann et al., 2013). These epigenetic modifications seem to have antagonistic effects as an enrichment of H3K4me3 in M cells and of H3K4me2 in BS cells is observed in several grass species (Danker *et al.*, 2008; Heimann *et al.*, 2013). Based on this evidence, it was proposed that a methyltransferase is recruited in a cell-specific way to convert low histone methylation states, such as HeK4me2, established by default in C_4_ *PEPC*, in HeK4me3 enabling promoter activation (Danker *et al.*, 2008). 

A few studies have identified unmethylated CpG islands in the C_4_ *PEPC* promoter (Langdale et al., 1991; Tolley et al., 2012). These regions, along with H3K4me3 may maintain an open chromatin state. Despite these CpG islands being unmethylated in both M and BS cells, a similar hypothesis regarding the recruitment of a methyltransferase has been proposed (Tolley *et al.*, 2012). This way, an open chromatin conformation is maintained, and transcription can be induced in M cells (Tolley *et al.*, 2012). Nevertheless, the identification and functional characterization of such methyltransferase(s) or de-methylase(s) is still to be carried out.

Although progress has been made over the last years towards a better understanding of the gene regulatory mechanisms underlying C_4_ *PEPC* cell-specific gene expression, there is still a lot more to be unveiled. More progress has been done regarding the characterization of important *cis*-elements than in the identification and characterization of key *trans*-factors regulating C_4_ *PEPC* cell-specificity. Although some TFs have been identified as binding to the C_4_ *PEPC* promoter and as putative regulators of C_4_ *PEPC* cell-specific gene expression, the key players are still missing. It is still to be identified the key TF or TFs that promote or impair C_4_ *PEPC* cell-specific gene expression. Therefore, we believe that more effort is necessary to identify new TFs regulating C_4_ *PEPC* gene expression and to understand the signalling pathways and the regulatory networks involved.

### Diel regulation

The circadian clock is an internal mechanism that regulates several biological processes, including C_4_ photosynthesis (Khan et al., 2010). Although the effects of the circadian clock on C_4_ *PEPC* gene expression remain largely unknown, a few studies have shown that similarly to other C_4_ genes, C_4_ *PEPC* gene expression has a circadian regulation (Horst et al., 2009; Khan *et al.*, 2010). C_4_ *PEPC* is an early morning phasing gene and, despite its light regulation, it presents an oscillatory rhythm under constant light (Horst *et al.*, 2009; Khan *et al.*, 2010; Xu et al., 2016).

In the maize C_4_ *PEPC* distal promoter region (1300 bps before ATG), some histone acetylation sites, such as H3K9ac, which has a high correlation with transcription activation, show circadian oscillation, maintaining its rhythmicity and high amplitude levels under constant light (Horst et al., 2009). These observations show that, though regulators of C_4_ *PEPC* cell-specific gene expression are located within the ﬁrst 500 bp upstream of the translational start codon (Gupta et al., 2020), the distal promoter region (1300 bps before ATG) might be more related to the C_4_ *PEPC* gene expression level, as well as with the circadian regulation.

It was shown that, during the night period of a diel cycle, histone acetylation is not totally removed (Offermann et al., 2006). These intermediary histone acetylation levels found during this period, contrast with the low acetylation levels found in this gene after a long period of dark exposure (Offermann *et al.*, 2006). Therefore, it was proposed that light regulates histone acetyltransferases (HATs), being also active under dark conditions to maintain steady-state acetylation levels (Offermann *et al.*, 2006). Therefore, one can hypothesise that HATs’ activity or expression levels may also be regulated by the circadian clock. Nevertheless, it was shown that high histone acetylation of the C_4_ *PEPC* promoter may not be enough to induce transcription. In maize, the treatment of darkened plant leaves with a histone deacetylase (HDAC) inhibitor did not alter C_4_ *PEPC* gene expression (Offermann *et al.*, 2006).

As described above, ZmbHLH80 and ZmbHLH90 participate in C_4_ *PEPC* regulation (Górska et al., 2019). Interestingly, in *Arabidopsis thaliana*, FBH1, a homologous TF to ZmbHLH80 and ZmbHLH90, is involved in the circadian rhythm regulation by repressing the *CCA1* gene expression (Nagel et al., 2014). FBH1 is also involved in the *CCA1* regulation in response to warm temperatures (Nagel *et al.*, 2014). It would be interesting to understand if this mechanism is conserved in maize, and other C_4_ species, and to unveil the regulators involved. This will help us to better understand how C_4_ *PEPC* and, eventually, other C_4_ genes are regulated by the circadian rhythm.

### Light regulation

Light is an important environmental stimulus regulating the genes involved in C_4_ photosynthesis, being C_4_ *PEPC* one of the C_4_ genes most responsive to light (Nelson et al., 1984; Schaffner and Sheen, 1992; Kausch et al., 2001; Offermann et al., 2006; Offermann *et al.*, 2008; Burgess et al., 2016; Xu et al., 2016). In greening assays, C_4_ *ZmPEPC* transcript level and promoter activity increase until several hours after illumination (Nelson *et al.*, 1984; Schaffner and Sheen, 1992; Kausch *et al.*, 2001; Xu *et al.*, 2016).

Despite the molecular mechanisms underlying C_4_ *PEPC* light regulation being still unclear, this gene is known to be light-regulated at different levels. In C_4_ *PEPC* distal promoter (between 3178 and 2908 bps before ATG) four cytosine residues were identified as differentially methylated in plants grown under different light conditions (Langdale et al., 1991; Tolley et al., 2012). These residues are less methylated in M cells of green leaves, compared with etiolated leaves or roots (Langdale *et al.*, 1991; Tolley *et al.*, 2012). In greening leaves, an increase in demethylation of two of these cytosine residues was also observed within 48h of light exposure (Langdale *et al.*, 1991). However, although the demethylation of these residues has a good correlation with the increase of C_4_ *ZmPEPC* transcript levels, it does not seem to be important for the cell-specific transcription of this gene, since its proximal promoter region is sufficient to drive M cell-specific expression (Tolley *et al.*, 2012; Gupta et al., 2020). Nevertheless, it is possible that upstream differentially-methylated regions can act as enhancers of C_4_ *ZmPEPC* expression in M cells, being their contribution to C_4_ *PEPC* expression still unclear (Tolley *et al.*, 2012).

In greening maize leaves, the chromatin of the proximal promoter region (500 bps before ATG) has an open state, compared with the chromatin of the same region in etiolated leaves, showing that light modulates chromatin dynamics of this region of C_4_ *PEPC* promoter (Kalamajka et al., 2003). In species from different C_4_ evolution origins, some histone acetylation sites in both coding and promoter regions of C_4_ *PEPC* are regulated by light (Table 1) (Offermann et al., 2006; Offermann *et al.*, 2008; Horst et al., 2009; Heimann et al., 2013). A comparison between both distal and proximal C_4_ *ZmPEPC* promoter regions revealed that acetylation levels have a stronger light response and higher correlation with transcription in C_4_ *ZmPEPC* distal promoter regions (Horst *et al.*, 2009). This further supports the idea that the distal promoter of C_4_ *PEPC* may contribute as an enhancer of C_4_ *PEPC* gene expression.

**Table 1 -:** Histone modifications found in C_4_ *PEPC* gene promoter and regulated processes.

Associated process	Modification	PEPC promoter region	Specie	Reference
Cell-specificity	H3K4me3 H3K4me2	Proximal	*Zea mays*	Danker et al., 2008
Circadian regulation	H3K9ac	Distal	*Zea mays*	Horst et al., 2009
Light regulation	H3K9ac	Proximal	*Zea mays* *Setaria italica*	Offermann et al., 2008; Horst et al., 2009; Heimann et al., 2013
		Distal	*Zea mays* *Sorghum bicolor*	
	H4K5ac	Proximal	*Zea mays* *Setaria italica*	Offermann et al., 2008; Horst et al., 2009; Heimann et al., 2013
		Distal	*Zea mays* *Sorghum bicolor*	
	H4K16Ac	Proximal and Distal	*Zea mays*	Horst et al., 2009
	H3K23Ac	Proximal and Distal	*Zea mays*	Horst et al., 2009

To control C_4_ *PEPC* acetylation levels, light modulates histone deactylases’ (HDACs) activity (Offermann et al., 2006; Offermann *et al.*, 2008). During the night period, some HDACs are activated to deacetylate the C_4_ *PEPC* promoter. During the day, although some HDACs are repressed, others are activated to maintain the steady-state histone acetylation levels (Offermann *et al.*, 2006; Offermann *et al.*, 2008). This shows that HDACs seem to be important to regulate the acetylation levels of C_4_ *PEPC*, however the HDACs involved in this regulation remain to be identified. It has long been known that light has an important role in modulating the binding of proteins to the C_4_ *PEPC* promoter (Kano-Murakami et al., 1991). *In vitro* experiments showed that nuclear factors extracted from green maize leaves are able to bind to the C_4_ *ZmPEPC* promoter, whilst the nuclear factors extracted from etiolated maize leaves are not. (Kano-Murakami *et al.*, 1991) A good example of a TF binding to the C_4_ *PEPC* promoter in a light-dependent manner is Dof1, whose activity is modulated by light (Yanagisawa and Sheen, 1998). Dof1 can induce higher C_4_ *PEPC* promoter activity in greening as compared with etiolated protoplasts (Yanagisawa and Sheen, 1998). Since both blue and red light induce the expression of C_4_ *PEPC*, it seems that both phytochrome and the cryptochrome pathways contribute to the regulation of C_4_ *PEPC* gene. However, the downstream players of this regulation remain to be unveiled (Hendron and Kelly, 2020). Being light an important stimulus regulating C_4_ *PEPC* expression, it would be interesting to identify and characterize more TFs that regulate C_4_ *PEPC* in response to light and unveil the regulatory mechanisms of the different photoreceptors.

Besides light playing a crucial role in regulating C_4_ *PEPC* gene expression, the signals originated from the interplay between light and chloroplast development seem to be relevant for C_4_ *PEPC* regulation (Kausch et al., 2001; Burgess et al., 2016). The inhibition of chloroplast development reduces the activation of the C_4_ *ZmPEPC* promoter and an increase in C_4_ *ZmPEPC* expression was observed in greening maize seedlings (Kausch *et al.*, 2001; Burgess *et al.*, 2016). Although one can hypothesise that chloroplast development is a relevant component of C_4_ *PEPC* gene expression regulation, the regulatory mechanisms are still unknown.

Despite being a crucial environmental cue regulating C_4_ *PEPC* gene expression, the regulatory mechanisms underlying light response need to be further investigated to better understand this topic. It would be interesting to unveil the regulatory mechanisms involved in the epigenetic modifications of C_4_ *PEPC* promoter in response to light and understand their relevance for C_4_ photosynthesis. The identification of TFs and *cis*-elements and downstream players of the different photoreceptor pathways involved in the regulation of C_4_ *PEPC* is also important for understanding the light regulatory networks. Finally, retrograde signalling is a rather unexplored topic regarding C_4_ *PEPC* expression. Since it seems to be a relevant component of C_4_ *PEPC* regulation, it would be important to understand the regulatory mechanisms involved in this process and the interplay between light and retrograde signalling.

## Response of C_4_ PEPC to adverse environmental conditions

Plants are sessile organisms that cannot escape from adverse environmental conditions. To cope with such conditions, plants need to re-arrange their metabolism. Photosynthesis is a key process for life on Earth, being essential for many different ecosystems. Alterations in this metabolic pathway can lead to serious decreases in plant yield, which is detrimental to our current agricultural systems. It is of utmost importance to understand how the adverse environmental conditions modulate the photosynthetic metabolism. Given the importance of C_4_ photosynthesis, it is particularly important to understand how this metabolism is affected by different environmental stresses. One of the key enzymes in C_4_ photosynthesis is C_4_ PEPC, but the mechanisms by which this protein is regulated under stress conditions remain unclear. Here we summarise the current knowledge regarding the effects of various stress conditions on C_4_ *PEPC* gene expression. Table 2 summarises the reported effects of different abiotic stresses on C_4_ PEPC levels.

**Table 2 -:** Summary of the abiotic stress effects in C_4_ PEPC levels.

Stress condition	Species	Regulatory effect	Reference
Osmotic stress	*Zea mays*	Decrease transcipt	Pelleschi et al., 1997; Foyer et al., 1998
		Increase activity	Ghannoum, 2009
	*Sorghum bicolor*	Decrease transcript	Buchanan et al., 2005
Salt stress	*Zea mays*	Increase activity	Hatzig et al., 2010)
	*Sorghum bicolor*	Increase transcript	Buchanan et al., 2005
Cold	*Zea mays*	Decrease activity	Selinioti et al., 1985; Angelopoulos and Gavalas, 1988; Chinthapalli et al., 2003
Heat	*Zea mays*	Increase activity	Crafts-Brandner and Salvucci, 2002; Chinthapalli et al., 2003
Nitrogen deficiency	*Zea mays*	Decrease transcript/protein	Sugiharto and Sugiyama, 1992; Sugiharto et al., 1992; Sugiharto *et al.*, 1990
Cadmium excess	*Zea mays*	Decrease activity	Wang et al., 2009
Ozone excess	*Zea mays*	Decrease transcript/protein	Leitao et al., 2007b ; Leitao *et al.*, 2007a

### Osmotic stress

Different adverse environmental conditions alter the osmotic balance within the cell, leading to osmotic stress. These conditions include for instance water deficit, salt stress (osmotic component), or osmolyte pressure (e.g. PEG-mediated drought). Although some studies have investigated the impact of osmotic stress in C_4_ plants it is still not clear its effect on the C_4_ cycle, with many authors claiming that the CBB cycle is the major limiting step in osmotic stress tolerance in C_4_ plants. 

Several reports have shown a decrease in C_4_ PEPC expression and activity in response to water deficit (Pelleschi et al., 1997; Foyer et al., 1998) but other authors have seen an increase of its activity under water deficit (Ghannoum, 2009). An increase in PEPC levels would raise the initial carboxylation of atmospheric CO_2_ and increase the carbon flux to BS. If not accompanied by an increase of Rubisco-mediated carboxylation, this increase would lead to decreased net carbon fixation, and subsequent CO_2_ leakage. Major effect of osmotic stress is the decrease of photosynthetic rate in both C_3_ and C_4_ plants. It has been proposed that, in C_4_ plants, an increase of non-used CO_2_ in the BS cells (i.e. ↑[CO_2_]_BS_) leads to CO_2_ leakage and subsequent decrease in net photosynthesis (Ghannoum, 2009), which could be linked with the changes in PEPC levels described in some works. 

Jeanneau et al., 2002 tested the effect of overexpression of *Sorghum bicolor* C_4_ *PEPC* in drought tolerance in maize. They observed an increase in carbon assimilation rates in lines with increased C_4_ *PEPC* expression and a decrease in the lines with decreased C_4_ *PEPC* expression, as it was expected. In terms of drought tolerance, no effect of the overexpression of C_4_ *PEPC* in severe drought conditions was observed, but plants showed a higher water use efficiency in mild-drought conditions. Together, C_4_ PEPC plays a role in regulating the carbon flux from M to BS cells, the increase of this flow may be beneficial in the early stages of drought but under more severe water deficit it becomes irrelevant. Overexpression of C_4_ PEPC alone seems to lead to an increase in transported CO_2_ that may not be efficiently used by Rubisco, either by Rubisco limitation or decarboxylation inefficiency, possibly due to a lack of increase in decarboxylation enzymes (e.g. NADP-ME).

Under salt stress, C_4_ plants showed higher PEPC activity contrary to C_3_ plants (Hatzig et al., 2010). There are no insights showing that this increase is linked to upregulation of photosynthesis but rather for the anaplerotic role of PEPC. It would be interesting to understand which component of the salt stress (osmotic or ionic) is indeed responsible for the upregulation of PEPC and which PEPCs are regulated at transcriptional level.

Work on *Sorghum bicolor*, analysed the genome wide transcriptional response to salt, PEG and ABA stress in both shoot and roots (Buchanan et al., 2005). In terms of C_4_ *PEPC* transcripts, it was observed an upregulation upon salt stress in both roots and shoots, which is in agreement with previous work in maize (Hatzig et al., 2010). PEG induced osmotic stress led to down regulation in roots but no changes in shoots, which is contrary to previous results in maize where either upregulation (Ghannoum, 2009) or downregulation (Pelleschi et al., 1997; Foyer et al., 1998) of C_4_ *PEPC* was observed. Abscisic acid treatment, a key hormone in stress response, leads to no change in *PEPC* transcript. 

Most genome wide studies in maize show no significant transcriptional response for C_4_ *ZmPEPC*, in both biotic and abiotic stresses [data obtained via Genevestigator (https://genevestigator.com/)].

### Temperature stress

High and low temperatures affect photosynthesis in both C_3_ and C_4_ plants. C_4_ plants are considered to be more sensitive to cold stress than C_3_ plants, due to the cold-labile feature of some C_4_ enzymes (Long, 1983). Plants that are more tolerant to low temperature usually show a higher accumulation of photosynthesis related enzymes, like Rubisco (Yamori et al., 2014). It was therefore expected that C_4_ plants under cold stress accumulated C_4_ related enzymes to counterbalance their reduced activity. Contrary to what was expected, C_4_ plants seem to show a decrease in PEPC activity under cold (Selinioti et al., 1985; Angelopoulos and Gavalas, 1988; Chinthapalli et al., 2003). It would be important to understand the transcriptional regulation and how knock-out or overexpression of C_4_ *PEPC* would affect temperature tolerance. 

Although cold decreases C_4_ PEPC activity, this effect is reversible when plants are placed back on optimal conditions. Though changes in activity its many times related to the phosphorylation of C_4_ PEPC, (Chinthapalli et al., 2003) showed that there are no changes in the phosphorylation status of C_4_ PEPC when treated with different temperature conditions, thus refuting the hypothesis of regulation by phosphorylation. The same study showed that C_4_ PEPC has increased activity at higher temperatures, in a way that is remarkably different from its C_3_ counterpart. On the other hand, (Crafts-Brandner and Salvucci, 2002) showed that C_4_ PEPC activity is rather insensitive to increase in temperature, although photosynthesis was reduced at temperatures higher than 40ºC. It would be important to investigate how different temperature conditions regulated C_4_ *PEPC* gene expression and how this correlates with photosynthesis efficiency. 

### Nitrogen levels regulation

Nitrogen deficiency is well known to cause a down regulation of C_4_ *PEPC* transcript and protein levels, in maize leaves (Sugiharto et al., 1990; Schlüter et al., 2012). On the other hand, upon nitrogen treatment, regardless of the form supplied (nitrate or ammonium), C_4_ *PEPC* transcript level and activity are significantly up regulated in maize (Sugiharto and Sugiyama, 1992; Suzuki et al., 1994). This up regulation is thought to be mediated by Glutamic acid, as its addition leads to an upregulation of the C_4_ *PEPC* gene expression and the inhibition of its synthesis leads to a down regulation (Sugiharto *et al.*, 1992). Nevertheless, the addition of ammonium does not affect the C_4_ *PEPC* gene expression in sorghum (Arias-Baldrich et al., 2017), indicating that regulation of C_4_ *PEPC* gene expression by nitrate or ammonium treatment may differ even among close C_4_ species. The fact that C_4_ *PEPC* gene expression can be modulated by nitrogen levels shows an intrinsic interplay between carbon and nitrogen metabolism, which may have been co-opted during C_4_ evolution. 

### Other stresses

It has been reported that cadmium affects the growth of maize plants by disturbing the light and carbon reactions of photosynthesis. High cadmium levels lead to a down regulation of C_4_ PEPC activity in maize, with the dosage affecting the time needed to see the effects (Wang et al., 2009). Whether this regulation takes place at the transcriptional level is not known.

Atmospheric conditions can also affect photosynthesis, namely the increase in ozone concentration. It has been shown that increase in atmospheric ozone led to impacts in maize growth and in its photosynthetic potential. Although the light harvesting complex is affected at relatively low increases of ozone, the carbon fixation reactions namely PEPC and Rubisco, are only affected at higher concentration with a reduction in protein amount and transcript (Leitao et al., 2007a , b). 

## Concluding remarks

During plant evolution, PEPCs evolved from bacterial PEPCs, after an ancestral duplication, when Viridiplantae arose. In C_3_ plants, PEPC is an important enzyme for plant development since it works as a link between carbon and nitrogen metabolism. Later, during C_4_ evolution, PEPC was recruited independently several times to incorporate the C_4_ cycle, by performing the first step of CO_2_ fixation. However, to obtain the features required for C_4_ photosynthesis operation, it was necessary to modify the mechanisms that regulate its gene expression, as well as protein accumulation and activity. Therefore, to engineer the C_4_ metabolism, it is crucial to understand the C_4_ PEPC regulatory network.

The regulation of C_4_ PEPC is complex, being modulated at several levels. At the epigenetic level, patterns of histone methylation were associated with the establishment of cell specificity. However, the mechanisms that maintain this pattern remain unknown. It would be interesting to investigate if there are methyltransferases recruited to the promoter in a cell-specific way, to induce higher levels of histone methylation, contributing to gene activation. If this is true, it would also be important to know which methyltransferases are recruited and the mechanisms underlying this process. Similarly, a deeper understanding of the role of CpG islands for the establishment of cell-specificity of C_4_ *PEPC* gene expression, would also be an interesting topic to investigate. Histone acetylation has been associated with light and circadian regulation and even not being crucial for C_4_ *PEPC* regulation, it may contribute. It would be interesting to investigate if histone acetylation can function as prerequisite to enable C_4_ *PEPC* transcription. In addition, it seems that different photoreceptors, may also be involved in C_4_ *PEPC* transcriptional regulation, since blue and red light induce C_4_ *PEPC* gene expression. In the future, it would be relevant to further characterise the regulatory mechanisms of C_4_ *PEPC* by the different photoreceptors, to better understand C_4_ *PEPC* light response. 

To establish cell-specificity, *cis*-elements and *trans*-factors were recruited during C_4_ evolution. Although some progress has been made to characterise C_4_ *PEPC* promoters and to identify putative regulatory *cis*-elements, there is still a gap regarding the identification and characterization of new *trans*-factors. It would be interesting to know which TFs bind to MEM1, a crucial *cis*-element defining cell-specificity in *Flaveria* species. In monocots, some TFs have been identified as putative regulators of cell-specificity. However, their relevance to establish cell-specificity and to C_4_ photosynthesis efficiency still needs to be demonstrated. The identification and characterization of key TFs to establish C_4_ PEPC cell-specificity in both monocots and dicots would be crucial to better understand these mechanisms. Furthermore, in both dicots and monocots, there are certainly relevant *cis*-elements in C_4_ *PEPC* gene promoter, involved in gene expression that remain to be identified.

The circadian regulation of C_4_ *PEPCs* is the most unexplored regulatory mechanism presented in this review. It is known that the circadian clock regulates C_4_ *ZmPEPC* at transcriptional level and its expression is regulated by ZmbHLH80 and ZmbHLH90. Since the Arabidopsis homologue for these two TFs, FBH1, regulates circadian clock through the transcriptional regulation of *CCA1*, it would be interesting to know if ZmbHLH80 and ZmbHLH90 could be involved in the circadian regulation of *ZmPEPC1* and if the regulation of *CCA1* is conserved.

Different species have distinct regulatory mechanisms to regulate developmental C_4_ *PEPC* gene expression and protein accumulation, which is not surprising, given that C_4_ photosynthesis is a convergent evolutionary event. Despite these differences, in all species, M cell differentiation seems to be important for a high C_4_ *PEPC* gene expression and protein accumulation. However, the regulatory mechanisms underlying leaf development are still poorly understood. In the future, it would be interesting to identify the internal cues involved in establishing M cell specificity along the developmental gradient.

The photosynthetic metabolism underpins the synthesis of carbohydrates needed for plant growth and reproduction. Adverse environmental conditions that negatively affect photosynthesis will impair plant growth and yield. It is therefore important to understand how photosynthesis responds to environmental stresses and find ways to improve such responses. In C_4_ photosynthesis, C_4_ PEPC plays an important role in carbon fixation, being responsible for the first carboxylation step in the cycle. Because of this role, C_4_ PEPC is tightly regulated and responds to environmental stimuli, such as water availability, light, nutritional signals, and atmospheric conditions. The regulation of C_4_ PEPC is poorly understood, but the effects of different environmental clues have been described. The regulation of C_4_ PEPC levels in response to stress is important to regulate the carbon flux into the C_4_ cycle, thus regulating the photosynthetic efficiency of the plant. It is difficult to distinguish between the role of C_4_ PEPC in the C_4_ cycle and its role in anaplerotic reactions. Being C_4_ PEPC an important enzyme for the C/N balance, its regulation can impact several metabolic pathways, making it a good target for improvement of plant stress response.

In conclusion, C_4_ evolution represents one of the most impressive cases of convergent evolution in Nature that has occurred independently over 60 times in very distant species. Nevertheless, their carbon concentration mechanisms always rely on a C_4_ PEPC, which is tightly regulated by internal and environmental cues. Since the function of C_4_ PEPC in C_4_ photosynthesis, combined with its anaplerotic role, makes it an important modulator of plant growth and yield, it is of utmost importance to better understand the gene regulatory network (including its evolution) modulating its expression and function.
